# Unbalanced diets enhance the complexity of gut microbial network but destabilize its stability and resistance

**DOI:** 10.1007/s44154-023-00098-x

**Published:** 2023-06-27

**Authors:** Penghao Sun, Mengli Wang, Wei Zheng, Shuzhen Li, Xiaoyan Zhu, Xuejun Chai, Shanting Zhao

**Affiliations:** 1 College of Veterinary Medicine, Northwest A&F University, Yangling, China; 2 College of Resources and Environment Sciences, Northwest A&F University, Yangling, China; 3 Aquatic EcoHealth Group, Fujian Key Laboratory of Watershed Ecology, Key Laboratory of Urban Environment and Health, Institute of Urban Environment, Chinese Academy of Sciences, Xiamen, China; 4 College of Basic Medicine, Xi’an Medical University, Xi’an, China

**Keywords:** Diet, Gut microbiota, Community stability, Co-occurrence network

## Abstract

Stability is a fundamental ecological property of the gut microbiota and is associated with host health. Numerous studies have shown that unbalanced dietary components disturb the gut microbial composition and thereby contribute to the onset and progression of disease. However, the impact of unbalanced diets on the stability of the gut microbiota is poorly understood. In the present study, four-week-old mice were fed a plant-based diet high in refined carbohydrates or a high-fat diet for four weeks to simulate a persistent unbalanced diet. We found that persistent unbalanced diets significantly reduced the gut bacterial richness and increased the complexity of bacterial co-occurrence networks. Furthermore, the gut bacterial response to unbalanced diets was phylogenetically conserved, which reduced network modularity and enhanced the proportion of positive associations between community taxon, thereby amplifying the co-oscillation of perturbations among community species to destabilize gut microbial communities. The disturbance test revealed that the gut microbiota of mice fed with unbalanced diets was less resistant to antibiotic perturbation and pathogenic bacteria invasion. This study may fill a gap in the mechanistic understanding of the gut microbiota stability in response to diet and provide new insights into the gut microbial ecology.

**Supplementary Information:**

The online version contains supplementary material available at 10.1007/s44154-023-00098-x.

## Introduction

Over the past decades, cumulative studies have well established the broad association and causality between gut microbiota and host health and illness risk (Morais et al. 2021). The gut is the primary location for the presence of host commensal microbiota, and with the development of biomics and bioinformatics, our understanding of the composition and function of gut microbes, microbe-microbe interactions, and microbe-host interactions continues to evolve (Valdes et al. 2018). The composition of the host gut microbiota is highly dynamic throughout the developmental stages of the host and mediates the maintenance of host physiological homeostasis and the onset and progression of disease (Fassarella et al. 2021). The colonization, growth, composition, and diversity of gut microbes are impacted by numerous factors, such as genotype, developmental stage, and environmental elements (Makki et al. 2018; Spor et al. 2011; Li et al. 2021). Among environmental factors, dietary habits have the most dramatic effect on the diversity and composition of the gut microbiota, and different dietary habits mold the gut microbial community in a time-related manner (Makki et al. 2018). Unbalanced diets, characterized by incompatibility with the nutritional requirements of the organism, have been linked to gut dysbiosis. For example, feeding mice a high-fat diet (HFD), known as the Western diet, can lead to dramatic dysbiosis, defined by the overgrowth of pathobionts and the reduction or complete loss of commensal bacteria (Christ et al. 2019). In addition, a persistent plant-based high refined-carbohydrate diet (HCD), particularly popular in Asia, induces metabolic syndrome associated with impaired gut permeability and microbiota dysbiosis (Ludwig and Ebbeling 2018; Hall et al. 2021). Although the effects of unbalanced diets, including HFD and HCD, on the composition of the gut microbiota have been recognized, their impact, particularly the underlying mechanisms, on the stability of the gut microbial community remains unclear.

Clarifying community structure and maintenance mechanisms is an important part of research on biodiversity and its response to perturbations. Microorganisms are not isolated; instead, they comprise a complex network of ecological interactions (Röttjers and Faust 2018). Therefore, network analysis has been widely used to study how environmental factors affect the stability of microbial communities (Röttjers and Faust 2018). In co-occurrence network analysis, individual taxa in a community are abstracted as network nodes, while edges are constructed based on the correlation of abundance between taxa. Finally, the properties of the microbial community are inferred by investigating the topological characters of the network. It has been shown that communities possessing specific network characters—higher modularity, fewer positive links between taxa, and increased negative links between taxa—are more robust (Coyte et al. 2015). Modularity is thought to reflect the heterogeneity of ecological niches, different selection mechanisms, and phylogenetic grouping of closely related populations, leading to non-random interaction patterns and ultimately to the complexity of the ecological network (Olesen et al. 2007). The high modularity of the network stabilizes the community by limiting the impact of the loss of a taxon on its own modules, thus preventing the extinction of that taxon from disrupting other parts of the network (Stouffer and Bascompte 2011). Meanwhile, theoretical work indicates that ecological networks dominated by negative interactions are more stable under disturbance (Neutel et al. 2002). This is because positively connected community members can respond to environmental fluctuations in tandem, resulting in positive feedback and co-oscillations that actually amplify the effects of disturbances on the community (Mougi and Kondoh 2012). In contrast, negative interactions may limit the dispersal of co-oscillations in the community and facilitate the stability of the network (Coyte et al. 2015). Despite this knowledge and the increasing utilization of network analysis, the symbiotic or potential interactions within the gut microbial community and the patterns of their responses to diet and the underlying mechanisms remain largely undiscovered.

The aim of this study was to investigate the effects of unbalanced diets on the stability of the gut microbiota using the mouse model. We hypothesized that the selective pressure resulting from unbalanced diets would reduce the gut microbial richness and cause the high niche overlap between existing taxa, reducing community modularity and negative:positive associations, thereby impairing community stability. Therefore, we first investigated whether unbalanced diets destabilized the stability of gut microbial community. We then investigated the effect of unbalanced diets on community assembly processes and how variations in community assembly processes affected microbial network properties, including modularity and taxa association. Finally, the antibiotic cocktail and invasive *Escherichia coli* (*E.coli*) were used to test the resilience of the gut microbiota to environmental perturbations. Our results showed that unbalanced diets reduced the stability of the microbial community stability and its resistance to perturbations. This study elucidated the adverse effects of unbalanced diets on the gut microbiota and provides new insights into the mechanistic understanding between dietary habits and gut ecology.

## Results

### Unbalanced diets disrupted the gut bacterial composition and reduced the bacterial richness

**Fig. 1:**
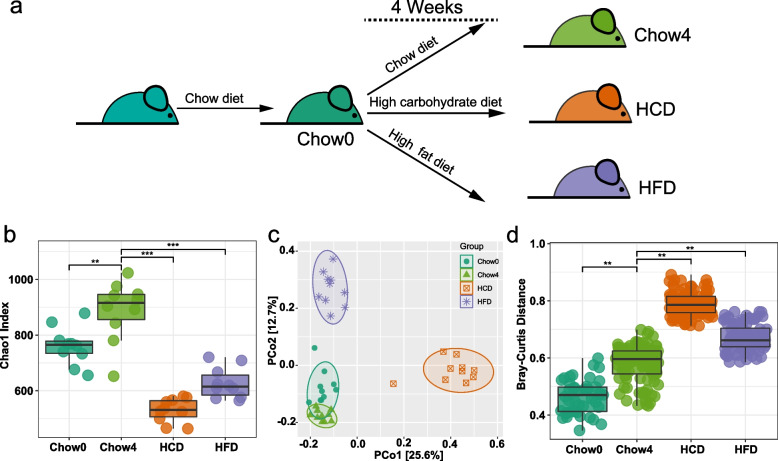
Response of bacterial communities to changes in dietary patterns. **a** Schematic representation of the animal experimental design (*n* = 10 for each group). **b** Alpha-diversity was represented by the Chao1 index. **c** Principal coordinate analysis (PCoA) showed differences in microbial composition. **d** Differences in beta-diversity among four groups were estimated based on a Bray–Curtis distance matrix. Lines in boxes represent median, top and bottom of boxes represent first and third quartiles, and whiskers represent 1.5 interquartile range. Statistical significance was determined by the Kruskal–Wallis test, adjusted for multiple comparisons by Dunn’s post-hoc. ** *p* ≤ 0.01, *** *p* ≤ 0.001

To elucidate the effects of unbalanced diets on microbial community stability, we subjected mice to a continuous plant-based high refined-carbohydrate diet (HCD) or high-fat diet (HFD) (Fig. 1a). Alpha diversity is the fundamental parameter to describe community characteristics and reveals the average species diversity at specific sites. In the present study, the Chao1 index was adopted to measure species alpha diversity, which represents the number of species in each sample. As shown in Fig. 1b, the gut microbial richness of standard chow-fed mice increased with host development. Meanwhile, principal coordinate analysis (PCoA) based on Bray–Curtis distance was performed to estimate the microbial beta diversity, and our result showed a separation in gut microbiota structure between Chow0 and Chow4 (Fig. 1c, d). These results indicate that the composition of the gut microbiota in mice varied with host development. Persistent unbalanced diets, including HCD and HFD, significantly reduced the alpha diversity of gut microbiota (Fig. 1b). We observed that the bacterial communities were significantly different between chow-diet and unbalanced-diet mice (Fig. 1b, c). Furthermore, we noted that the gut microbial community of HCD mice was dominated by the first principal coordinate, whereas the second principal coordinate was the main driver of gut bacterial characteristics in HFD mice (Fig. 1c), suggesting that the filtering effect of diet on gut microbiota depended on diet composition. The intricate relationship between microbial diversity and community stability encouraged us to investigate the systematic response of gut microbiota under different diets.

### Unbalanced diets enhanced the complexity of gut microbial network but destabilized its stability

**Fig. 2:**
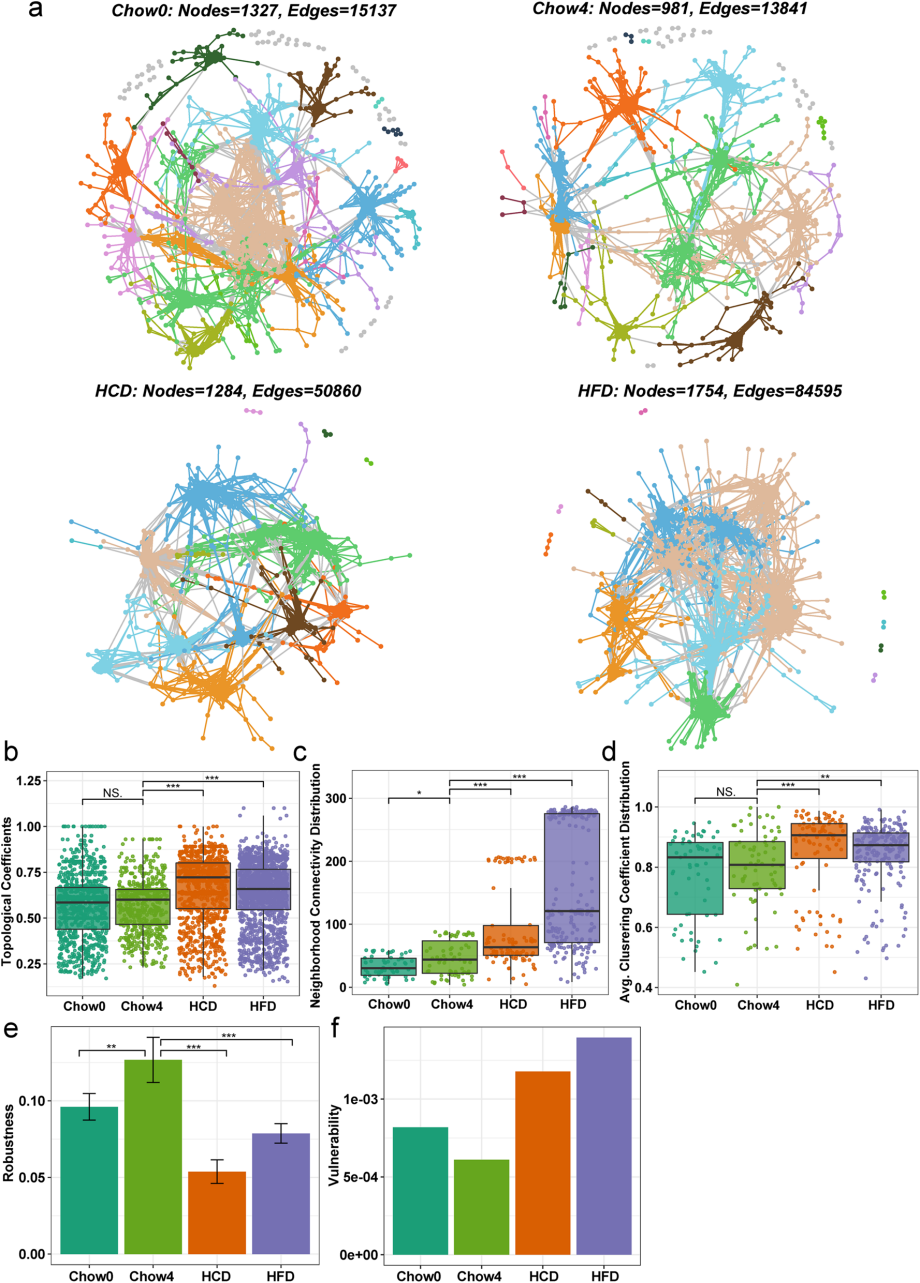
Complexity and robustness analysis for microbial networks. **a** Visualization of constructed microbial networks. Nodes represent individual amplicon sequence variants (ASVs) and are filled with color by module attributes. Dietary shifts affect the complexity of microbial networks, including topological coefficients (**b**), neighborhood connectivity (**c**), and average clustering coefficient (**d**), as well as stability, including robustness (**e**) and vulnerability (**f**). Lines in boxes represent median, top and bottom of boxes represent first and third quartiles, and whiskers represent 1.5 interquartile range. In **e**, bars indicate group mean ± SD. In **b**-**d**, *p* values were determined by Kruskal–Wallis with Dunn’s multiple comparison test. In **e**, *p* values were determined by one-way analysis of variance (ANOVA) with Dunnett’s multiple comparison test. NS Not Significant, * *p* ≤ 0.05, ** *p* ≤ 0.01, *** *p* ≤ 0.001

We constructed separate microbial co-occurrence networks for each group based on Spearman correlations between ASVs and determined their properties. Compared to Chow0, the microbial network of Chow4 comprised fewer nodes and edges, indicating a convergent simplicity of associations between gut microbiota during host development (Fig. 2a). Meanwhile, persistent standard diet exerted a more negligible impact on the properties of nodes (Chow0 VS Chow4), including topological coefficients and average clustering coefficient, except for neighborhood connectivity (Fig. 2b-d). In contrast to the standard diet, persistent unbalanced diets increased the size of the network (Fig. 2a). Furthermore, we found that the topological coefficients, neighborhood connectivity, and average clustering coefficient of network nodes increased significantly by 110.2% to 116.6%, 198.3% to 352.2%, and 106.0% to 107.1%, respectively, in the mice with unbalanced diets compared to those in Chow4 mice (Fig. 2b-d). These results suggest that unbalanced diets significantly increased the complexity of the gut microbial network.

The stability of microbial networks was tested by removing nodes to change the amplitude of natural connections (Fig. 2e) or by deleting single nodes from the network to measure the maximum decrease in network efficiency (Fig. 2f). Based on random node loss, the robustness of the microbial network was significantly lower in the mice with unbalanced diets than in the standard diet-fed mice (Fig. 2e). Network vulnerability was also higher in the unbalanced diet-fed mice than in the standard diet-fed mice (Fig. 2f). These results indicate that unbalanced diets destabilized the stability of gut microbiota. Interestingly, the gut microbial network in Chow4 mice exhibited higher robustness and lower vulnerability compared to that in Chow0 mice (Fig. 2e, f), demonstrating that the gut microbiota network becomes more stable as the host develops.

### Modularity and taxa association influenced network stability

**Fig. 3:**
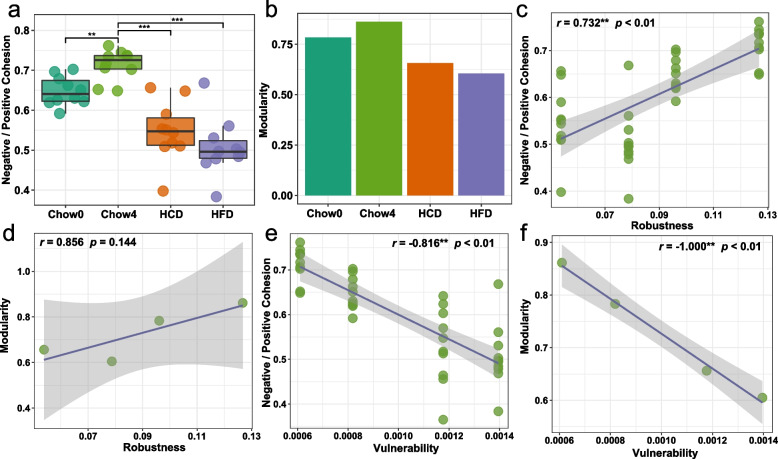
Modularity and association among taxa affected the stability of network. Unbalanced diets decreased the ratio of negative:positive cohesion in gut microbiota (**a**) and the modularity of gut microbial networks (**b**). The robustness of microbiota network was positively correlated with the ratio of negative:positive cohesion (**c**) and modularity (**d**). The vulnerability of the microbiota network was negatively correlated with the ratio of negative:positive cohesion (**e**) and modularity (**f**). In **c**-**f**, smoothing curves based on the linear model are shown in gray with 95% confidence intervals. The correlation is determined by Spearman analysis. Lines in boxes represent median, top and bottom of boxes represent first and third quartiles, and whiskers represent 1.5 interquartile range. In **a**, *p* values were determined by one-way ANOVA with Dunnett’s multiple comparison test. ** *p* ≤ 0.01, *** *p* ≤ 0.001

Network properties, in particular modularity and taxa association, have been used to predict network stability successfully (Hernandez et al. 2021). Communities connected by positive links are typically considered unstable. The cohesion method provides insight into associations between species resulting from positive and negative taxa interactions through disentangling distinct positive and negative co-occurrence (Herren and McMahon 2017). As shown in Fig. 3a, the gut microbiota of mice fed with unbalanced diets exhibited a lower ratio of negative:positive cohesion, which was positively correlated with robustness (*r* = 0.732, *p* < 0.01) and negatively correlated with vulnerability (*r* = -0.816, *p* < 0.01) of gut microbial networks (Fig. 3c, e). Networks with high modularity showed more limited shifts in composition in response to environmental perturbations, and thus possess higher stability (Stouffer and Bascompte 2011). Modularity analysis showed that the gut microbial network of unbalanced diet-fed mice showed lower modularity compared to that of chow-fed mice (Fig. 3b). Consistent with previous studies, network modularity was positively but insignificantly correlated with robustness (*r* = 0.856, *p* = 0.144) and significantly negatively correlated with vulnerability (*r* = -1.000, *p* < 0.01) of gut microbial networks (Fig. 3d, f). These results suggest that the decrease in gut microbiota stability was, at least in part, due to the reduction in network modularity and the decrease in negative:positive cohesion.

### Phylogenetic conservation of bacterial responses to unbalanced diet as drivers of decreasing negative:positive cohesion and modularity

**Fig. 4:**
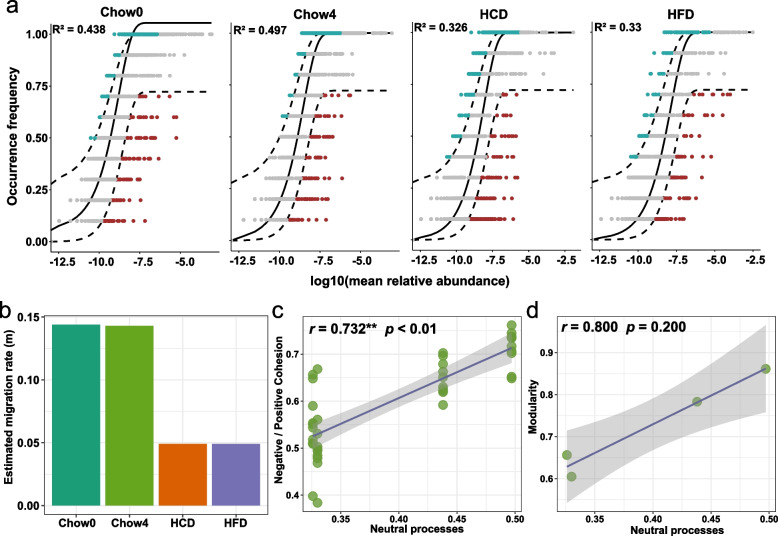
Deterministic process decreased the negative:positive cohesion and modularity. **a** The predicted occurrence frequencies for Chow0, Chow4, HCD, and HFD. ASVs that occur more frequently than predicted by the model are shown in blue, while those that occur less frequently than predicted are shown in red. Dashed lines represent 95% confidence intervals around the model prediction (black line). **b** The estimated migration rate for gut bacterial communities. Neutral processes were positively correlated with the ratio of negative:positive cohesion (**c**) and modularity of networks (**d**). The correlation is determined by Spearman analysis. In (**c**-**d**), smoothing curves based on the linear model are shown in gray with 95% confidence intervals. ** *p* ≤ 0.01

An open question is how dietary patterns affect microbial network properties, which are highly correlated with community stability. The occurrence and intensification of selection pressure reduce the frequency of negative interactions and enhance the effectiveness of positive interactions within the community (Hammarlund and Harcombe 2019). Diet is thought to be the strongest selective pressure on gut microbes, mediated in part by nutrient suitability (Fassarella et al. 2021). To investigate the impact of dietary patterns on the community assembly processes of the gut microbiota, the neutral community model was performed. Our results showed that the fit of the model was decreased in HCD (0.326) and HFD (0.33) mice compared to that in Chow4 (0.497) (Fig. 4a). Notably, the effect of neutral processes on community assembly increased with host development (Chow0 VS Chow4) (Fig. 4a). The estimated migration rates were higher in mice with standard diet than that in unbalanced diet-fed mice (Fig. 4b), suggesting that unbalanced diets increased dispersal limitation in gut microbial communities. These results reveal that persistent unbalanced diets increased the selective pressure on the gut microbial community. Correlation analysis revealed a significant positive correlation between the fit of the neutral model and the ratio of negative:positive cohesion (*r* = 0.732, *p* < 0.01) and a positive but non-significant correlation with the modularity of the network (*r* = 0.800, *p* = 0.200) (Fig. 4c, d), demonstrating that unbalanced diet-induced selective pressure decreased the negative:positive cohesion between taxa and the modularity of networks, which contributed to destabilizing the microbial network stability.

**Fig. 5:**
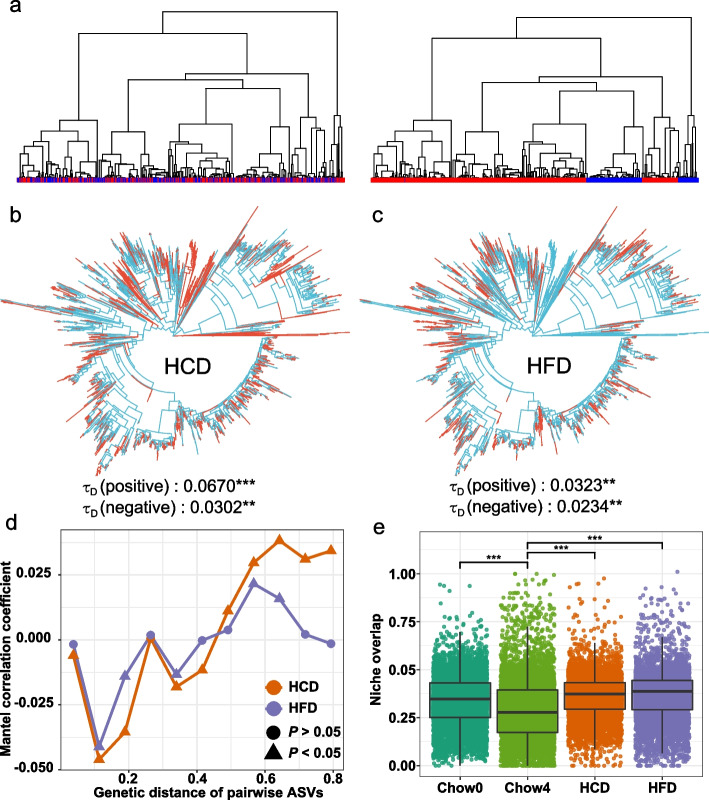
Phylogenetic conservation of bacterial responses to unbalanced diet. **a** The conceptual framework for the phylogenetic conservation analysis. Bacterial taxa respond positively (red) or negatively (blue) to perturbation. The responses might be phylogenetically random (left) or conserved (right). The bacterial response to HCD (**b**) or HFD (**c**) was conserved phylogenetically (red: positive response, blue: negative response). **d** Mantel correlograms showing the difference in response magnitude versus genetic distance of pairwise ASVs. **e** The degree of overlap of ecological niches was assessed by Levins index. The statistical significance of phylogenetic conservation of diet response was determined by permutation test (999 times). In (**d**), we relate between-ASV differences of response magnitude to between-ASV phylogenetic distances using Mantel correlograms with permutation-based significance tests (999 times). In (**e**), *p* values were determined by one-way ANOVA with Dunnett’s multiple comparison test. ** *p* ≤ 0.01, *** *p* ≤ 0.001

Recent studies have shown that microbial responses to environmental perturbations are always phylogenetically conserved (Isobe et al. 2019), and result in high ecological niche overlap of resident species, which enhances positive interactions in the community (Faust and Raes 2012; Michalska-Smith et al. 2022). Thus, elucidating the nature of the bacterial response to diet will aid our understanding of the mechanisms underlying changes in community stability. DESeq2 method was adopted to quantify the response of bacterial ASVs to unbalanced diets (red: positive response, blue: negative response) (Fig. 5b, c). We further performed consenTRAIT analysis to examine whether the bacterial response to unbalanced diets was phylogenetically conserved. The mean genetic depth (τD) for both positive and negative responses ranged from 0.0234 to 0.0670, and the permutation test indicated that the bacterial response to unbalanced diets was significantly associated with phylogeny. In addition to the direction of the responses, the magnitude of microbial response to unbalanced diets was also phylogenetically structured (Fig. 5d). Notably, the gut microbes of HFD mice showed a significant phylogenetic signal limited to short phylogenetic distances. Phylogenetic correlogram analysis showed that the response magnitude of any pair of ASVs was significantly negatively correlated with the short pair’s genetic relatedness. At very high genetic distances, correlations became positive, capturing synergistic responses across deeper clades. Ecological niche overlap was assessed using the Levins index, and we found that persistent unbalanced diets significantly enhanced ecological niche overlap in the gut microbiota (Fig. 5e). These results well explained the increased proportion of positive cohesion in mice fed with unbalanced diets.

### Unbalanced diets reduced the resistance of gut microbiota to environmental perturbations

**Fig. 6:**
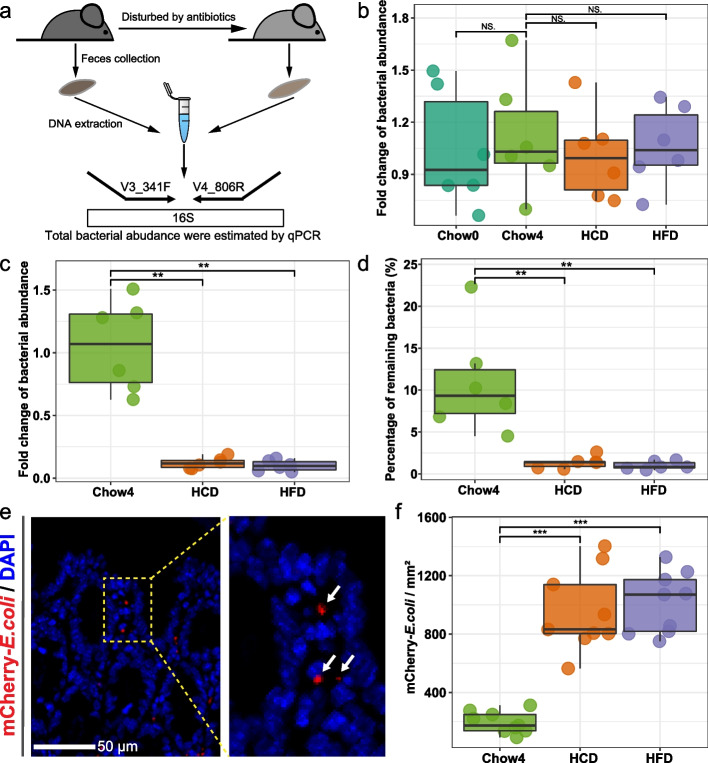
Unbalanced diets destabilizing the stability and resistance of gut microbiota. **a** Schematic representation of antibiotic disturbances (*n* = 6 for each group). **b** Fold-change in total bacteria in fecal samples from four groups. **c** Effect of antibiotic cocktails on the amounts of total bacteria in fecal samples, as shown by fold change. Total bacterial amounts were quantified by qPCR amplifying universal bacterial 16S rRNA genes (V3-V4). **d** The percentage of remaining bacteria in fecal sample after antibiotic disturbances. **e** Fluorescence microscopy of *E.coli* (red) in colonic tissue. Nuclei were counterstained with DAPI (blue). *n* = 9 slices from 3 mice. **f** The density of mCherry-*E.coli* in the colon section. Lines in boxes represent median, top and bottom of boxes represent first and third quartiles, and whiskers represent 1.5 interquartile range. *P* values were determined by one-way ANOVA with Dunnett’s multiple comparison test. NS Not Significant, ** *p* ≤ 0.01, *** *p* ≤ 0.001

To further assess the stability of gut microbial communities, mice were treated with antibiotic cocktail to evaluate the resistance of gut microbiota to disturbances (Fig. 6a). Fecal samples prior to antibiotic treatment were collected, and bacterial abundance was determined by *q*PCR to eliminate the effect of raw microbial abundance on perturbation experiments. No significant difference was found in the amounts of total bacteria in fecal samples among the four groups (Fig. 6b), suggesting that host development and unbalanced diets exhibited weak effects on fecal bacterial abundance. Consistent with the results of network stability analysis (Fig. 2e, f), mice fed with unbalanced diets exhibited lower bacterial abundance and percentage of remaining bacteria in the fecal sample after antibiotic disturbances relative to mice with normal diet (Chow4) (Fig. 6c, d). A stable intestinal environment reduces the risk of infection by inhibiting pathogen colonization. To assess the resistance to pathogen invasion, mice were gavaged with mCherry-*E.coli* and sacrificed after eight hours (Fig. 6e). Consistent with the above results, adherence of *E.coli* to the mucosal surface of the colon was increased in mice fed unbalanced diets, including HCD and HFD, compared to that in chow-fed mice (Chow4) (Fig. 6f). These results suggest that unbalanced diets destabilized the resistance of gut microbiota to environmental perturbations.

## Discussion

Understanding the relationship between dietary habits and gut microbial response is essential for gut ecosystem management. The adverse effects of unbalanced diets on individuals are widely recognized (Sonnenburg and Bäckhed 2016). However, little is known about the impact of unbalanced diets on gut microbiota stability, which plays a crucial role in mediating the communication between gut microbes and host health. In the present study, we found that the gut microbiota of mice fed with unbalanced diets was less diverse, less modular, and dominated by positive cohesion than that of mice with the standard diet, which contributed to destabilizing community stability. Perturbation experiments showed that unbalanced diets reduced the resistance of gut microbiota to antibiotic disturbances and pathogen invasion. Below, we discuss how these results have extended our knowledge of how unbalanced diets disrupted the stability of gut microbiota and the underlying mechanisms.

### Unbalanced diet-induced selective pressure affected network modularity and taxa associations

Dietary habits shape diverse gut microbiota profiles, an important reason being the differences in nutritional ecological niches brought by food components (Byndloss et al. 2018). For instance, persistent dietary patterns, especially the consumption of proteins and animal fats (*Bacteroides*) versus plant carbohydrates (*Prevotella*), cause specific enrichment patterns in the gut microbiota, which are referred to as enterotypes (Wu et al. 2011). In addition, a higher Firmicutes/Bacteroides ratio has been observed in human populations of westernized regions (T. C. Liu et al. 2021b). These findings reveal the tight association between dietary habits and gut microbial composition. Selection occurs when differences in fitness and ecological niches among taxa lead to microorganisms' proliferation or death at different speeds. Since the colonization of the microbial community in the gut ecosystem is closely related to the availability and accessibility of nutrients in that context, the molding effect of food components on gut microbes is considered to be a selective pressure (Fassarella et al. 2021; Foster et al. 2017).

**Fig. 7:**
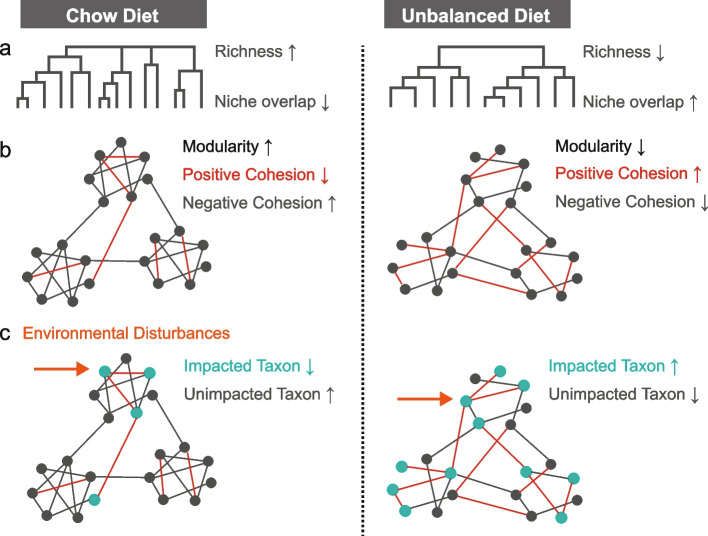
The proposed mechanism for unbalanced diet destabilizes the stability of gut microbiota. **a** Unbalanced diet decreased the bacterial richness and increased niche overlap among existing species. **b** The phylogenetically conserved responses of gut microbiota to diet reduced the network modularity and enhanced the positive cohesion between taxa. **c** These increased more positive feedback loops between taxa when community members face environmental disturbances, resulting in reduced community stability

Recent studies well established that microbial responses to environmental stress are always phylogenetically conserved, implying that the response of closely related bacterial taxa to disturbances is more similar (A. C. Martiny et al. 2013). Thus, researchers concluded that phylogenetic information about bacterial responses could be used to predict the response of uncharacterized but phylogenetically related taxa (J. B. Martiny et al. 2015). We found that the response of the gut microbiome to diet-induced selective pressure is significantly phylogenetically conserved (Fig. 5b, c). Environmental filtering plays an essential role in shaping communities, favoring a subset of species with similar functional traits (*e.g*., adaptations to diet composition) that promote colonization under these conditions (Ortiz et al. 2021). The phylogenetically conserved response increases the overlap of ecological niches of existing species under stress (Fig. 7) (Morrissey et al. 2016; Amend et al. 2016). Within the context of species interactions, positive correlations may result from synergistic cooperation between taxa for environmental resource utilization (Faust and Raes 2012). Thus, the phylogenetically conserved response to dietary selection pressure resulted in the increased positive association among taxa in communities (Fig. 3a), which are more easily altered in composition in response to disturbances and are hard to return to baseline.

Modules are assumed to be groups of highly interconnected species in a community that potentially construct a biological pathway that links the individual to the overall emergence properties of the network (Dubin et al. 2016; McHardy et al. 2013). Modularity quantifies the extent to which taxa are classified as co-occurring taxa. Species that share similar niches tend to be organized into the same community modules (Allesina and Levine 2011). Thus, modules are considered to be response units to disturbance. Our results showed that unbalanced diet-induced selective pressure reduced the modularity of co-occurring networks (Fig. 4d). The phylogenetically conserved response of gut microbiota to unbalanced diets leads to high niche overlap among taxa, and thus existing species tend to form more uniform modules while reducing the modularity of the network (Fig. 7).

### Decreased the negative:positive cohesion and modularity as processes destabilizing gut microbial communities

Clarifying the relationship between diet and microbial community stability will help us to better understand the negative effects of unbalanced diets on individuals. Nevertheless, microbial network stability in response to unbalanced diets remains largely elusive. To determine whether unbalanced diets impair the stability of the constructed network, we employ a variety of indicators to assess network stability, involving robustness and vulnerability. The pioneering work by Dunne et al. suggests that network robustness can be measured by the proportion of the remaining nodes in the network after randomly deleting nodes (Dunne et al. 2002). The vulnerability of each node is defined as the relative contribution of that node to the efficiency of the entire network (Deng et al. 2012). Network vulnerability is represented by the maximum vulnerability of the nodes in the system. In the present study, we found that unbalanced diets impaired the stability of gut microbiota, including a decrease in network robustness and an increase in vulnerability (Fig. 2e, f).

The ability of microbial communities to maintain stability or restore from disruptions relies on their diversity and the intricate interactions between community taxa (Flint et al. 2017; Coyte et al. 2015). Previous studies have shown that positive correlations in communities may result from facilitation between species, while negative correlations may represent the result of species competition (Durán et al. 2018; Zelezniak et al. 2015). Under stress, gut microbiota commonly adopts cooperative traits to maintain competitiveness within a community (Fassarella et al. 2021). Microorganisms utilize quorum-sensing signaling molecules that serve as a communication system, informing the network density, dispersal conditions, and composition of the microbiota in the surrounding environment, allowing the microbiota to collectively shift their behavior in response to environmental modifications (Papenfort and Bassler 2016). Positive interactions in microbial communities reinforce positive feedback loops. The impact of perturbations on the community is exacerbated by the fact that perturbations experienced by any one member of these loops can be rapidly propagated throughout the entire system (Coyte et al. 2015). In contrast, negative interactions can lead to negative feedback loops that dampen not only perturbations experienced by their own members, but also those experienced by linked positive feedback loops (Fig. 7) (Coyte et al. 2015). Cohesion analysis revealed that unbalanced diets enhanced the intensity of positive interactions among taxa (Fig. 3a), which was negatively correlated with community stability (Fig. 3C, E), suggesting that the decline in community stability is, at least in part, attributable to the alteration in member interactions. Furthermore, theoretical expectations suggest that increased complexity, but lower modularity, is associated with decreased network stability (Gravel et al. 2016). One advantage of modularity is that fluctuations in species within a module are less likely to spread to taxa in other modules because of the limited connectivity between them (Fig. 7) (Stouffer and Bascompte 2011). We noted that unbalanced diets reduced the gut bacterial richness (Fig. 1b) but increased the co-occurrence network size (Fig. 2a), suggesting that the reduced modularity of gut microbial network in mice with unbalanced diets may be attributed to the increase in the frequency of taxa interactions.

### The linkage between community stability of gut microbiota and host health

The human commensal microbiota comprises hundreds of species and trillions of cells that reside primarily in the gastrointestinal tract (Gill et al. 2006). These microorganisms provide numerous health benefits to the host, including the catabolism of complex compounds in food, defense against pathogens, and maturation of mucosal immunity (Sun et al. 2022). The gut microbes are frequently concerned due to its ecological stability. Without perturbation, the gut microbial community presents a dynamic equilibrium by oscillating around a stable ecological condition (Relman 2012). Populations from different lifestyle traditions may harbor distinct microbial species, while individuals tend to carry the same core species over time (Ganguli et al. 2019). The stability of the commensal flora is deemed essential for the health of the host, as it ensures that the relevant functions beneficial to the host are maintained over the long term (Fassarella et al. 2021). Thus, disturbances in intestinal flora homeostasis may cause or exacerbate disease development.

Stability and resistance are essential ecological characteristics of the gut microbiota (Lozupone et al. 2012). According to dietary recommendations, a balanced diet is a prerequisite for human health (Bechthold et al. 2018). Greater microbiota stability has protective consequences for human hosts by decreasing the risk for localised or systemic infections (Lloyd-Price et al. 2016). The regulation of the human gut microbiota by dietary interventions has been widely investigated. In ecological terms, diet habit is one of the most important selection pressures on the gut microbiota, partly caused by competition for nutrients (Byndloss et al. 2018). The increased intake of western diets is thought to have chosen a microbiota with altered composition and function, consisting of the loss of some carbohydrate-degrading species (Sun et al. 2022). Although several studies have shown that high-carbohydrate diet promotes longevity and improves mid-life and early late-life cardiometabolic health, current public health advice recommends reducing the intake of ultra-processed carbohydrates because of their association with gut microbiota-mediated metabolic disease (Wali et al. 2021). However, the effects of dietary composition on the stability of the intestinal ecosystem are largely unrevealed. Our findings fill a gap in the understanding between dietary habits and the subsequent impacts on gut microbial stability.

A healthy gut environment is typified by a high degree of microbial diversity, which facilitates functional biodiversity and microbial-host interactions (Le Chatelier et al. 2013). Higher microbial diversity leads to elevated functional redundancy, which is generally regarded as contributing to the stabilization of microbiota function during perturbations (Le Chatelier et al. 2013). In the present study, our results revealed that the gut bacterial diversity of mice on a persistent standard diet was enhanced with host development, suggesting that the gut microbiota tends to be highly diverse as the host develops. In contrast, persistent unbalanced diets reduced the bacterial diversity of gut microbiota (Fig. 1b). Microbial communities with high diversity show greater resistance to invasion by non-native bacteria and expansion of opportunistic pathogens, leading to the phenomenon referred to as colonization resistance (Buffie and Pamer 2013). In addition, there is evidence that the initial composition of the gut microbiota clearly determines the impact of the disturbance on the ecosystem (Raymond et al. 2016). It appears that a lower initial microbiota diversity benefits the enrichment of opportunistic pathogens (Raymond et al. 2016). These results suggest that dietary habits not only affected the structure of the gut microbes, but also determined the outcome of microbial response to perturbations.

Although a stable microbial community is necessary for host health, the effect of diet on individuals is often a holistic effect and not limited to a particular microenvironment. Therefore, oversimplifying the relationship between microbiota variance and diet effects on host health would be inaccurate. For example, very low carbohydrate, high-fat ketogenic diets are recommended for the treatment of a variety of diseases, including cardiovascular diseases and central nervous system diseases (Mardinoglu et al. 2018). At the same time, researchers also found that this diet pattern can reduce the diversity of gut microbiota and inhibit the abundance of specific probiotics, including *Lactobacillus* and *Bifidobacterium* (Yoo et al. 2021). In addition, although the World Health Organization recommends a reasonable dietary structure, the definition of a healthy diet remains a challenge (Blüher 2019). Therefore, the relationship between dietary habits and host health needs to be clarified by in-depth research.

## Conclusion

In conclusion, our study provides the first evidence that persistent unbalanced diets impaired bacterial community stability to environmental perturbations. The underlying mechanism is that unbalanced diets increase the selection pressure on the gut flora, which increases positive associations in the system and decreases community modularity, creating more positive feedback loops between taxa to destabilize microbial stability. Since the stability of gut microbiota is considered essential for host health, elucidating how diet affects the gut microbial community will contribute to deepening the understanding of the interactions between diet and host physiological homeostasis and the ecology of gut microbes.

## Methods

### Mice, diets and experimental setup

Male ICR mice were obtained from the Experimental Animal Center of Xi'an Jiaotong University, China. Mice were housed in groups of 3–4 mice per cage in an animal facility under standard conditions (12-h light/dark cycle, humidity of 50 ± 15%, and temperature of 22 ± 2 °C). Standard pelleted diet and unbalanced diets were purchased from Jiangsu Xietong Medicine Bioengineering Co., Jiangsu, China, and kept at -20 °C for the duration of the study. Prior to the dietary intervention, mice were fed conventional chow (Chow diet). 4-weeks-old mice were randomly divided into three groups (*n* = 10) to receive the following diets respectively for four weeks: A) Chow4 (Chow diet); B) HCD (high refined-carbohydrate diet which consisted of 80.3% kcal from carbohydrates); C) HFD (high fat diet which consisted of 64.6% kcal from fat). Notably, the mice of the Chow0 group were consistent with those of the Chow4 group. See Supplementary Table 1 for details on the structural composition of the diet. Animal janitors and investigators performing the experiments were blinded to the group assignment of mice during the experiment.

### Fecal sample DNA extraction and PCR amplification and sequencing

Fecal samples of 4-week-old mice belonging to the Chow4 group were collected as Chow0. Feces from the other three groups (Chow4, HCD, and HFD) of mice were collected four weeks after the dietary intervention. The mice were placed in sterile cages and waited for their spontaneous defecation, and the faeces were picked up with sterile forceps. All samples were frozen at -80 °C before DNA extraction and analysis. QIAamp DNA Stool Mini Kit (QIAGEN, Germany) was used to extract fecal bacterial DNA, and all procedures were based on the manufacturer’s instructions. The V3-V4 hypervariable regions of the bacterial 16S rRNA gene were amplified with primers 341 F (5’-CCTAYGGGRBGCASCAG -3’) and 806 R (5’-GGACTACNNGGGT ATCTAAT-3’). DNA extraction and subsequent sequencing steps were conducted by Magigene Technology Co., Ltd. Guangzhou, China. The concentration of the extracted DNA needs to be measured at no less than 25 ng/3μL prior to PCR amplification.

### Processing of sequencing data

Sequencing data were processed following our previous protocols (Sun et al. 2022). In brief, raw bacterial 16S rRNA gene sequence data were generated by Illumina Miseq PE250. After truncating barcodes and primers, the sequence data were imported into the Quantitative Insights Into Microbial Ecology2 (QIIIME2) platform for further analysis (Bolyen et al. 2019). Divisive Amplicon Denoising Algorithm 2 (DADA2) was applied for these sequences denoising, generating representative sequence and amplicon sequence variants (ASVs) table. The taxonomy of each representative bacterial gene sequence was analyzed by the RDP Bayes-Classifier using a confidence threshold of 80%.

### Antibiotic disturbances

In order to examine the stability of gut microbiota, a wide-spectrum antibiotic cocktail was added in the drinking water for mice lasting three days at the following concentration: ampicillin (1 g/L), clindamycin hydrochloride (0.5 g/L), and streptomycin (1 g/L) (Takahashi et al. 2020). This antibiotic cocktail is prepared fresh on the day of treatment. Faeces were collected from each group of mice prior to the antibiotic cocktail treatment and again three days later from each group of mice. To check the effects of the three antibiotics on the gut microflora, qPCR using bacterial DNA in fecal samples was conducted following an improved published protocol (Takahashi et al. 2020). Universal primers for bacterial 16S rDNA synthesized by Tsingke biotechnology co., Ltd (Beijing, China) were used: 341 F (5’-CCTAYGGGRBGCASCAG -3’) and 806 R (5’-GGACTACNNGGGT ATCTAAT-3’).

### Generation of ampicillin-resistant mCherry-*E. coli*

*E. coli* MG1655 was purchased from Feng Hui Sheng Wu Co., Hunan, China, and transformed with the plasmid PUC57-Tac-mCherry to induce the expression of red fluorescent protein and β-lactamase conferring resistance to 100 mg/ml of ampicillin.

### Quantitative analysis of mCherry-*E. coli* in intestinal mucosa

Eight hours after gavage with mCherry-*E. coli* (10^8^ cfu/ml, 10 ml/kg), mice were sacrificed, and the colonic tissue was harvested. Colonic tissue was fixed with 4% paraformaldehyde. The immunofluorescence procedures were performed following our previous protocols (Sun et al. 2022).

### Statistical information

For the 16S rRNA gene amplicon sequencing data, a rarefied ASVs table at 16866 reads per sample was created to calculate bacterial diversity by the ‘microeco’ package in R (C. Liu et al. 2021a). A neighbour-joining phylogenetic tree was inferred using the phylogeny plugin of QIIME2 (Bolyen et al. 2019). In the figure legends, each significance test is described in detail, including post-hoc analysis.

To estimate species coexistence of gut microbiota, co-occurrence networks were constructed following the published protocol (de Vries et al. 2018). In briefly, robust correlations with Spearman’s correlation coefficients (*r*) > 0.6 and false discovery rate-corrected *p*-values < 0.01 were used to construct networks. To describe the topology of the networks, we calculated a set of metrics: topological coefficients, neighborhood connectivity, and average clustering coefficient. To verify the interactions strengths of the bacterial community, we also calculated cohesion, which is an abundance-weighted, null model-corrected metric based on pairwise correlations across taxa, by referring to Yuan et al. (Yuan et al. 2021):

$$\text{cohesion }=\sum_{i=1}^{m} {\text{ abundance }}_{i}\times {\text{ connectedness }}_{i}$$

where *m* is the total number of taxa in a community. Higher absolute values of Cohesion represent stronger species interactions.

The robustness of the network was defined as the proportion of the remaining species in this network after random node removal. To test the effects of species removal on the remaining species, we calculated the abundance-weighted mean interaction strength (wMIS) of node *i* according to the published method, *i.e*. (Yuan et al. 2021):

$${\text{wMIS }}_{i}=\frac{\sum_{j\ne i} {b}_{j}{s}_{ij}}{\sum_{j\ne i} {b}_{j}}$$

where *b*_*j*_ is the relative abundance of species *j* and *s*_*ij*_ is the association strength between species *i* and *j*, which is measured by the Pearson correlation coefficient. After removing the selected nodes from the network, if wMIS_*i*_ = 0 or wMIS_*i*_ < 0, node *i* was considered extinct/isolated and thus removed from the network. This process continued until all species had positive wMISs. The proportion of the remaining nodes was reported as the network robustness. We measured the robustness when 50% of random nodes or five-module hubs were removed (Yuan et al. 2021). The vulnerability of each node measures the relative contribution of the node to the global efficiency. The vulnerability of a network is indicated by the maximal vulnerability of nodes in the network (Yuan et al. 2021).

To determine the community assembly processes of gut microbiota, we used a neutral community model to predict the relationship between ASV detection frequency and their relative abundance across the wider metacommunity (Burns et al. 2016). In this model, *m* is an estimate of the migration rate. The parameter *R*^2^ represents the overall fit to the neutral model. Calculation of 95% confidence intervals around all fitting statistics was done by bootstrapping with 1000 bootstrap replicates.

To assess whether the bacterial response to diet was phylogenetically conserved, we applied consenTRAIT analysis, which followed the methods of Martiny et al. (A. C. Martiny et al. 2013). A positive (log_2_-fold ratio > 0) or negative (log_2_-fold ratio < 0) response was assigned for each ASV on the phylogenetic tree based on the log_2_-fold ratio exported from DESeq2. To estimate τ_D_, we first identified the root node of clades where at least 90% of the members shared the trait. We then estimated the average consensus sequence distance (*d*) between the root node (*R*_*j*_) of n clades (*j*) sharing a given trait and the *m* members (*i*) of clades (that is, the leaves, *S*_*i*_):

$${\tau }_{\mathrm{D}}=\frac{1}{n}\sum_{j}^{n} \frac{1}{m}\sum_{i}^{m} d\left({S}_{i}j\right)$$

This estimate was repeated for each bootstrap tree. We attributed the presence of singleton entries to undersampling and included a non-parametric estimate for significant clustering by randomly assigning traits 1000 times (10 times to each bootstrap tree) to entries in the phylogenetic tree as a null distribution. Then we compared the estimated τ_D_ to this null distribution to assess the statistical significance of the bacterial response.

## Supplementary Information

**Additional file 1:** **Supplementary Table 1.** Diet designs for experiments.

## Data Availability

All data in this manuscript are available on reasonable request to the corresponding authors. The 16S rRNA gene sequencing data have been deposited at the National Center for Biotechnology Information (NCBI) database under the accession PRJNA874363 (https://www.ncbi.nlm.nih.gov/sra/PRJNA874363). Computer code used in this study is available upon request from the corresponding authors.

## Abbreviations

HFD: High-fat diet
HCD: High refined-carbohydrate diet
*E.coli*: *Escherichia coli*
PCoA: Principal coordinate analysis
τD: The mean genetic depth;
ASV: Amplicon sequence variant
ANOVA: One-way analysis of variance
