# Supplementary material for: Unbalanced diets enhance the complexity of gut microbial network but destabilize its stability and resistance
Source: Stress Biol. 2023 Jun 27;3(1):20. doi: 10.1007/s44154-023-00098-x (PMC10441997; doi:10.1007/s44154-023-00098-x)
Supplement: Supplementary file 1 — Additional file 1: Supplementary Table 1. Diet designs for experiments. [file 44154_2023_98_MOESM1_ESM.docx]

**Supplementary Table 1. Diet designs for experiments**

| Macronutrient content (% kcal) | Chow | HFD | HCD |
| --- | --- | --- | --- |
| Protein | 22.4 | 14.7 | 14.7 |
| Carbohydrate | 59.6 | 20.7 | 80.3 |
| Fat | 18 | 64.6 | 5 |
|  |  |  |  |
| Ingredients (g/kg) |  |  |  |
| Casein | 200 | 140 | 140 |
| L-Cystine | 3 | 1.8 | 1.8 |
| Corn Starch | 330 | 0 | 680 |
| Maltodextrin | 117.5 | 80.9 | 80.9 |
| Sucrose | 0 | 240 | 0 |
| Cellulose | 140 | 50 | 50 |
| Soybean | 162 | 0 | 0 |
| Lard | 0 | 440 | 0 |
| Mineral Mix | 35 | 35 | 35 |
| t-Butylhydroquinoe | 0.01 | 0.05 | 0.05 |
| Vitamin Mix | 10 | 10 | 10 |
| Choline chloride | 2.5 | 2.5 | 2.5 |
